# Shoseiryuto Ameliorated TDI-Induced Allergic Rhinitis by Suppressing IL-33 Release from Nasal Epithelial Cells

**DOI:** 10.3390/pharmaceutics14102083

**Published:** 2022-09-29

**Authors:** Manabu Kitano, Seiya Fukuoka, Naoki Adachi, Tadashi Hisamitsu, Masataka Sunagawa

**Affiliations:** Department of Physiology, School of Medicine, Showa University, 1-5-8 Hatanodai, Shinagawa-ku, Tokyo 142-8555, Japan

**Keywords:** Shoseiryuto, TDI-induced allergic rhinitis, IL-33 release

## Abstract

Toluene diisocyanate (TDI) is a major cause of occupational asthma and rhinitis. Shoseiryuto (SST) is one of the traditional herbal medicines (Kampo medicine) and has long been used as a natural medicine for allergic diseases such as allergic rhinitis (AR) and asthma. Recent studies have shown that the expression and release of IL-33, which regulates the T_H_2 cytokine response in epithelial cells, is an important step in developing the inflammatory response of the nasal mucosa. In this study, we investigated whether SST may ameliorate the TDI-induced AR-related symptoms in rats and inhibit IL-33 release from nasal epithelial cells. An AR rat model was generated by sensitization and induction with TDI. SST was administered during the sensitization period. AR-related symptoms in rats were evaluated, and IL-33 release was measured both in vivo and in vitro. SST suppressed symptoms appearing in TDI-induced AR model rats, such as elevated serum histamine and IL-33 levels in nasal lavage fluid (NLF)/serum, which were suppressed by SST administration. TDI-induced IL-33 release from the nasal epithelial cell nuclei was also observed and suppressed in SST-treated rats and cultured nasal epithelial cells. These results suggest that SST ameliorates the symptoms of TDI-induced AR at least partially by inhibiting IL-33 release from nasal epithelial cells.

## 1. Introduction

Allergic rhinitis (AR) is induced by allergens such as ragweed and other pollens, mites, and fungi. It is one of the most common allergic inflammatory diseases, affecting more than 600 million people worldwide [1,2]. The immune response in the nasal cavity can be divided into two phases: an early IgE-dependent response and a late T_H_2 cytokine-dependent response [2,3,4]. Early-phase reactions are symptoms such as rhinorrhea and sneezing that occur within 30 min of allergen exposure, whereas late-phase reactions are described as congestion and fatigue that occur up to 24 h [2].

Airway epithelial cells provide an important barrier function in the superficial layer of the airways, preventing the entry of harmful substances from the environment. In addition to this barrier function, the airway epithelium also plays a role in controlling inflammatory conditions by producing and secreting cytokines and chemokines that regulate various inflammatory responses [5].

Among the inflammatory cytokines involved in the late phase of AR, IL-33 has been highlighted as one of the key cytokines. IL-33 belongs to the IL-1 family and activates the same intracellular signaling pathway as IL-1 and IL-18, with ST2 as a receptor [2,6,7]. It has been suggested that IL-33 may induce allergic inflammation by inducing T_H_2 cytokine production in various immune cells, including T_H_2 and mast cells [2,8]. Importantly, Haenuki and colleagues revealed that IL-33 knockout mice showed significant reductions in the frequency of sneezing, total IgE levels and IgE-related responses, and infiltration of eosinophils and basophils into the nasal mucosa after ragweed challenge [2].

IL-33 protein is expressed in the nuclei of nasal epithelial cells in the normal condition and is rapidly released into the nasal fluid after exposure to allergens [2,9]. In humans, it has also been shown that IL-33 protein level was markedly reduced in nasal epithelial cells of the patients with AR compared to healthy controls [2].

Toluene diisocyanate (TDI) is a leading cause of occupational asthma (OA) and is also known to induce allergic rhinitis [5]. Regarding TDI-induced immune responses, induction of interleukins (ILs), including IL-4, 13, 25, and 33, in human bronchial epithelial cells has been reported [5].

Shoseiryuto (SST), called Xiao-quinglong-tang in China and So-cheong-ryong-tang in Korea, is one of the traditional herbal medicines (Kampo medicine) that has long been used as a natural medicine for allergic diseases, such as AR and asthma. SST has been used to improve both acute symptoms, including sneezing and rhinorrhea, and chronic symptoms, including nasal obstruction, in AR patients [10]. In relation to the mechanisms underlying the effects of SST, it has been reported that SST inhibited histamine release from mast cells in rats [11] and decreased the serum concentration of IgE AR patients [12]. Furthermore, it has been reported that SST improves the symptoms of nasal mucosa in a rat AR model by suppressing the transcription of mRNAs of T_H_2 cytokines, including IL-4, 5, and histamine H1 receptor (H1R) [9].

In the present study, we show an ameliorative effect of SST on TDI-induced AR symptoms, which has not yet been clarified, and examined the relationship between the effect of SST and the IL-33 release from nasal epithelial cells.

## 2. Materials and Methods

### 2.1. TDI-Induced Rat Model of AR

Sprague-Dawley (SD) male rats at the age of 5 weeks (120–130 g) were purchased from Nippon Bio-Supp. Center (Tokyo, Japan). Three or 4 rats were housed in a cage in the conditioned room (12:12 h light/dark cycle at 25 °C ± 1 °C, with 45% ± 5% humidity). The rats accessed food (CLEA Japan, CE-2, Tokyo, Japan) and water ad libitum. All efforts were made to minimize animal suffering and to reduce the number of animals used. This study was conducted with the approval of the Committee of Animal Care and Welfare of Showa University and in accordance with the guidelines established with the Committee of Animal Care and Welfare of Showa University (certificate number: 07064; date of approval: 1 April 2017). Each experiment was repeated two times.

### 2.2. Sensitization and Provocation of Animals

**Figure 1:**
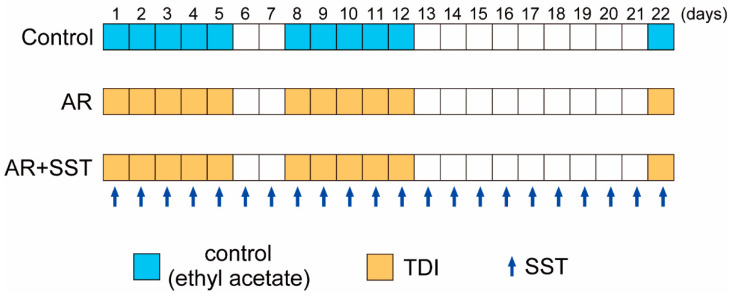
The experimental schedule to develop an allergic rhinitis (AR) rat model and clarify the effect of SST on AR development. The AR and AR + SST groups were sensitized by dropping 5 μL of 10% TDI dissolved in ethyl acetate and olive oil into each nostril for 5 consecutive days. The rats of the Control group were treated with intranasal ethyl acetate and olive oil instead of TDI in the same protocol. After 2 days of rest, the rats were resensitized in the same way for 5 days. Ten days after the second course of sensitization, the rats were provoked by intranasal administration of 5 μL of 10% TDI. SST was administered to the AR + SST group rats from the first day of the initial sensitization until the day of the final challenge with TDI (a total of 22 days).

Eighteen rats were randomly assigned to tree groups. Control: distilled water; Allergic rhinitis (AR): TDI; AR + SST: TDI + SST. The AR and AR + SST groups were sensitized with a modified protocol of Zheng et al. [13] by dropping 5 μL of 10% TDI (89870; Sigma-Aldrich, St. Louis, MO, USA) dissolved in a 1:4 mixture of ethyl acetate and olive oil (FUJIFILM Wako Pure Chemical Co., Osaka, Japan) into each nostril for the 1st 5 days. Control group rats were treated with an intranasal mixture of ethyl acetate and olive oil instead of TDI. After 2 days of rest, the rats were resensitized in the same way for 5 days. Ten days after the 2nd sensitization, the rats were provoked with an intranasal dose of TDI (5 μL of 10% TDI). SST was administered to the AR + SST group rats from the 1st day of the initial day of sensitization until the day of the last provocation with TDI (a total of 22 days; Figure 1).

### 2.3. Preparation of SST

SST (Lot. 2140019010) was kindly gifted by Tsumura and Co. (Tokyo, Japan) and prepared using the same procedures as previously reported [14]. Five grams of dried SST powder was prepared from extraction with boiling water of 8 herbs: 6.0 g of Pinellia tuber (*Pinellia ternata*, Breitenbach), 3.0 g of Asiasarum root (*Asiasarum sieboldi,* F. Maekawa), 3.0 g of Cinnamon bark (*Cinnamomum cassia*, Blume), 3.0 g of Ephedra herb (*Ephedra sinica* Stapf), 3.0 g of Glycyrrhiza (*Glycyrrhiza uralensis*, Fischer), 3.0 g of Schisandra fruit (*Schisandra chinensis* Baillon), 3.0 g of Paeony root (*Paeonia lactiflora*, Pallas), and 3.0 g of Processed ginger (*Zingiber officinale*, Roscoe).

### 2.4. Behavioral Assay

#### 2.4.1. Evaluation of Nose Scratching and the Number of Sneezes

After the last TDI stimulation, each animal was observed for 10 min, and the number of sneezes and the time spent nose scratching were scored.

#### 2.4.2. Nasal Lavage Fluid and Blood Collection

Nasal lavage fluid (NLF) was collected from rats anesthetized with intraperitoneal administration of pentobarbital sodium (50 mg/kg; Somnopentyl, Kyoritsu Seiyaku Co., Tokyo, Japan). The trachea was incised, a polyethylene catheter with a diameter of 1 mm was inserted into the nasopharyngeal cavity, and 1 mL of PBS flushed from the nostrils was collected. The NLF was centrifuged at 400× *g* for 10 min, and the supernatant was collected, stored at −80 °C, and used for measurement.

Subsequently, blood was collected from the inferior vena cava after laparotomy.

### 2.5. ELISA for NLF and Serum IL33

IL-33 concentrations in NLF and serum were examined in triplicate using an ELISA test kit (SEB980Ra; Cloud-Clone, Houston, TX, USA) according to the manufacturer’s protocol.

### 2.6. Preparation of Serum and Histamine Determination

Blood was centrifuged at 400× *g* for 10 min. The serum was withdrawn, and the histamine content was measured using an ELISA kit (A05890; Bertin Bioreagent, Montigny-le-Bretonneux, France) according to the manufacturer’s protocol.

### 2.7. Immunohistochemistry

After sampling NLF, the anesthetized rats were perfused intracardially with cold PBS (pH 7.4) and then perfused with 4% paraformaldehyde/PBS. The tissue, including the nasal cavity, was excised and cut into 20 μm thickness by a cryostat (CM1860; Leica Biosystems, Wetzlar, Germany). Then immunostaining process was conducted: rinse with PBS 3 times, blocking (10% goat serum with 0.3% Triton X-100 in PBS) for 2 h at room temperature, incubation with primary antibody (rabbit anti-IL-33, 1:200, bs-2633R, Bioss, Woburn, MA, USA) for 48 h, washing with PBS 3 times, incubation with 2nd antibody (donkey anti-rabbit Alexa Fluor 555, 1:1000, #A31572, Thermo Fisher Scientific, Waltham, MA, USA). Nuclei were counter-stained with DAPI (4′,6-diamidino-2-phenylindole, 1:1000, Thermo Fisher Scientific). Immunofluorescent images were obtained with a confocal microscope (FV1000D, Olympus, Tokyo, Japan).

### 2.8. TDI-HSA Preparation

TDI-HSA was prepared as reported previously [15]. Briefly, 2.4 g of TDI was reacted with 90 mL of 1% human serum albumin (HSA)/PBS with constant stirring for 30 min. To terminate the reaction, 2 M ammonium carbonate was added to the solution. As for control-HSA, 1% HSA solution was used. To remove unreacted TDR, reacted TDI-HSA and control-HSA solutions were centrifuged (3000× *g*, 20 min). Then, TDI-HSA and control-HSA solutions were dialyzed with a dialysis tube (Spectra/Por, MWCO 12-14,000, Repligen, Waltham, MA, USA) for 3 days with 4 L of 0.1 M ammonium carbonate. TDI-HSA and control-HSA were precipitated with an equal volume of 20% trichloroacetic acid and then redissolved with 1 M sodium hydroxide. The solution was dialyzed again (overnight, 3 times with 4 L of distilled water). Synthesized TDI-BSA was visualized by Coomassie blue staining after native SDS-PAGE.

### 2.9. Cultured Nasal Epithelial Cells and Immunocytochemistry

Human Nasal Epithelial Cells (HNEpCs) (Promocell, Rockville, MD, USA) were cultured on poly-L-lysine coated cover glasses (Matsunami, Osaka, Japan) in Airway Epithelial Cell Growth Medium (Promocell) and maintained in 5% CO_2_ and 95% air at 37 °C. Twelve hours after stimulation with TDI-HSA, HNEpCs were fixed with 4% sucrose-containing 4% paraformaldehyde (Sigma Chemical Co.) for 20 min. The fixed cells were permeabilized with 0.2% Triton X-100/PBS (Sigma Chemical Co.) for 5 min and blocked with 10% goat serum/PBS for 1 h. Then, an anti-IL-33 antibody (Nessy-1, 1:250, Enzo Life Sciences, Farmingdale, NY, USA) was applied overnight at 4 °C. IL-33 was visualized by isotype-specific secondary antibody conjugated with Alexa 488 (1:200, Molecular Probes, Eugene, OR, USA). A fluorescent microscope (Axio Observer, Carl Zeiss, Oberkochen, Germany) was used to obtain fluorescent images.

### 2.10. Statistics

All the values are expressed as means and standard error of the mean (SEM). Each data set was firstly analyzed using the Kolmogorov–Smirnov test to determine whether the data were normally distributed or not. One-way ANOVA followed by Tukey’s multiple comparisons or Kruskal-Wallis tests was used for normally distributed or non-normally distributed data, respectively, using EZR (Saitama Medical Center, Jichi Medical University, Saitama, Japan) [16]. *p*-value < 0.05 was considered statistically significant.

## 3. Results

### 3.1. SST Suppressed Symptoms Appeared in TDI-Induced AR Model Rats

Rats were sensitized to/provoked with TDI and administered SST in the schedule, as shown in Figure 1.

**Figure 2:**
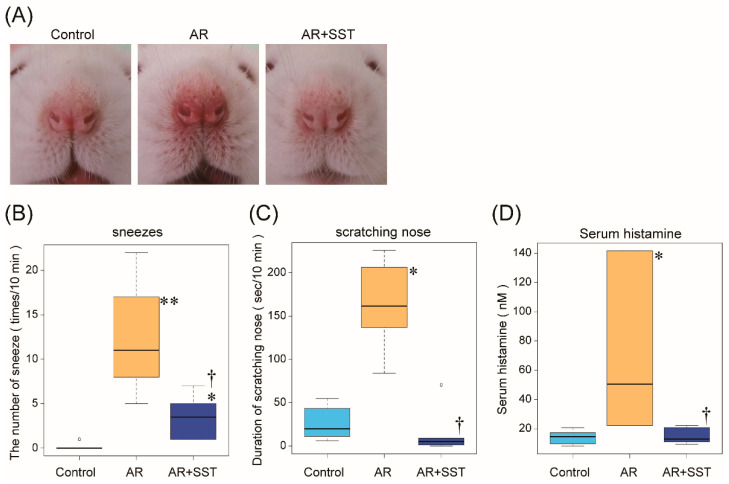
SST suppressed symptoms associated with AR. (**A**) Nose appearance of TDI-sensitized rats. Note, redness and swelling of nose appeared in AR rat is subsided in SST-treated rat. (**B**) The number of sneezes during 10 min immediately after the last TDI-induced (ethyl acetate for control) provocation. (n = 6) * *p* < 0.05, ** *p* < 0.01 vs. Control group; † *p* < 0.05 vs. AR group. (Kruskal-Wallis tests) (**C**) Duration of scratching nose for 10 min after the last TDI (ethyl acetate for control) provocation. Values are the mean ± SEM. (n = 6) * *p* < 0.05 Control group; † *p* < 0.05 vs. AR group. (Kruskal-Wallis tests) (**D**) Serum histamine concentration of rats. Values are the mean ± SEM. (n = 6) * *p* < 0.05 Control group; † *p* < 0.05 vs. AR group. (One-way ANOVA followed by Tukey’s multiple comparisons).

Provocation with TDI after sensitization to the reagent clearly induced apparent allergic symptoms such as reddening and swelling in the nose of rats (Figure 2A). Rats treated with SST, however, had a similar nasal appearance to control rats (Figure 2A).

Next, the number of sneezes and duration of nose scratching for 10 min immediately after provocation were determined. In TDI-sensitized AR rats, the total number of sneezes was 12.3 ± 2.58 while 0.17 ± 0.17 in the control rats (Figure 2B). Administration of SST significantly suppressed sneezing (3.50 ± 1.02; Figure 2B). SST also reduced the increased duration of scratching nose observed in AR rats (162 ± 20.7 s in AR rats; 15.1 ± 11.2 s in AR + SST rats; Figure 2C).

### 3.2. Elevation of Serum Histamine in TDI-Sensitized AR Rats Was Inhibited by SST Administration

Serum histamine levels were also measured as a marker of inflammatory levels in rats. As expected, serum histamine levels were dramatically raised in TDI-sensitized rats, which was suppressed by SST treatment (14.3 ± 2.04 nM in control rats; 71.5 ± 22.8 nM in AR rats; 15.0 ± 2.19 nM in AR + SST rats) (Figure 2D). These results clearly showed that SST treatment could protect rats against sensitization and provocation induced by TDI.

### 3.3. IL-33 Concentrations in NLF/Serum Were Increased in TDI-Sensitized AR Rats, Which Was Suppressed in SST-Treated Rats

**Figure 3:**
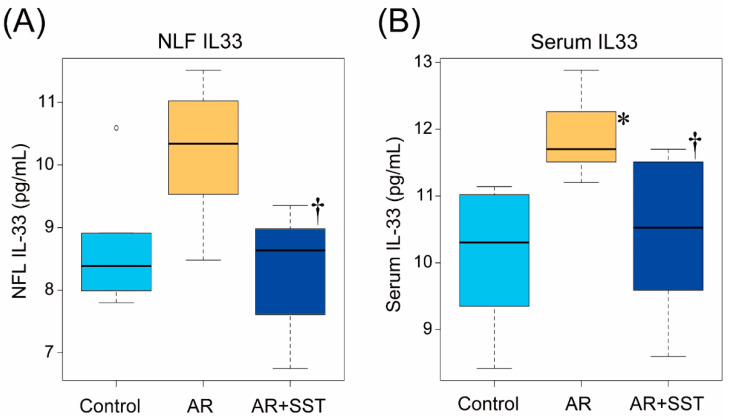
SST inhibited the increase of IL-33 concentrations in NLF and serum. (**A**) IL-33 concentration in NLF. (n = 6) † *p* < 0.05 vs. AR group. (One-way ANOVA followed by Tukey’s multiple comparisons). (**B**) IL-33 concentration in serum. Values are the mean ± SEM. (n = 6) * *p* < 0.05 Control group; † *p* < 0.05 vs. AR group (One-way ANOVA followed by Tukey’s multiple comparisons).

We next determined IL-33 levels in NLF and serum. TDI-sensitized AR rats showed significantly elevated IL-33 levels in serum and a trend toward elevation in NLF, while control rats had similar levels of IL-33 in both serum and NAL as control rats. (Figure 3A) (8.68 ± 0.41 in control rats; 10.2 ± 0.44 in AR rats; 8.33 ± 0.39 pg/mL in AR + SST rats). In parallel with the NLF IL-33, serum IL-33 also increased in AR rats, and SST treatment prevented the increase after TDI-sensitization. (Figure 3B; 10.1 ± 0.43 in control rats; 11.9 ± 0.25 in AR rats; 10.4 ± 0.48 pg/mL in AR + SST rats).

### 3.4. TDI-Induced IL-33 Release from the Nuclei of Nasal Epithelial Cells Was Inhibited in SST-Administered Rats

**Figure 4:**
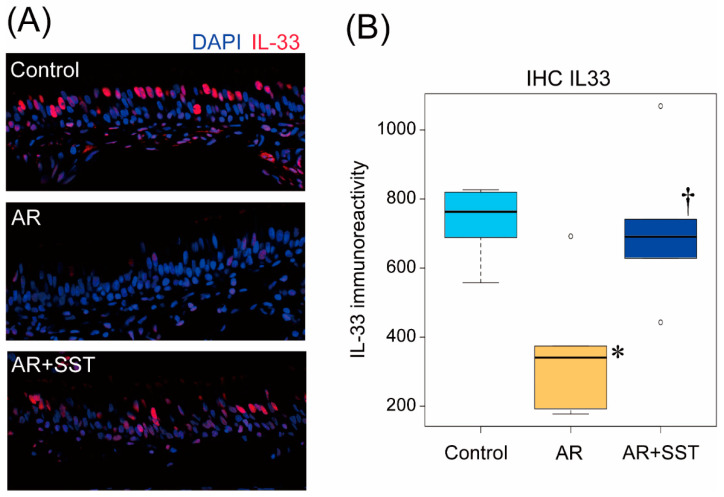
SST inhibited the release of IL-33 from nasal epithelial cells induced by the TDI-induced provocation. (**A**) Representative images of mucosal tissue from the nasal cavity stained with anti-IL-33 antibody. Red: IL-33, Blue: DAPI. Bar = 50 μm (**B**) Quantified data for immunoreactivity of IL-33 in the tissue. (n = 5) * *p* < 0.05 Control group; † *p* < 0.05 vs. AR group. (One-way ANOVA followed by Tukey’s multiple comparisons).

Immunohistochemical analysis showed that IL-33 expression in the nucleus of nasal epithelial cells in TDI-sensitized AR rats was dramatically reduced, whereas stable nuclear expression of IL-33 was observed in nasal epithelial cells of control rats (Figure 4A,B). Interestingly, SST treatment significantly restored the decrease in nuclear expression of IL-33 in nasal epithelial cells despite TDI sensitization/provocation (Figure 4A,B).

### 3.5. SST Suppressed TDI-Induced IL-33 Release from Cultured Nasal Epithelial Cells

**Figure 5:**
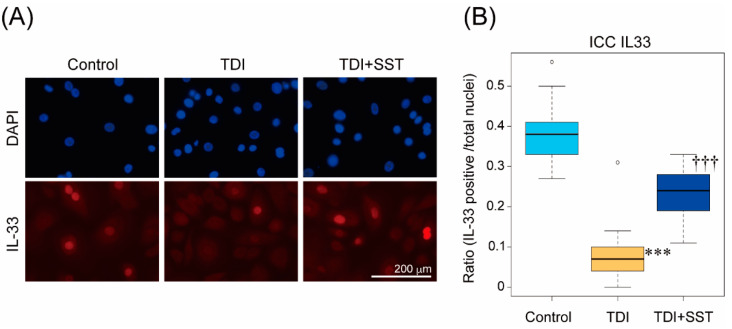
SST suppressed the TDI-induced IL-33 release from the nucleus of cultured nasal epithelial cells. (**A**) Representative images of cultured human nasal epithelial cells stained with anti-IL-33 antibody. Green: IL-33, Blue: DAPI. Bar = 200 μm (**B**) Quantified data of the ratio (IL-33 positive/total nuclei). (n = 22) *** *p* < 0.001 Control group; ††† *p* < 0.001 vs. AR group. (One-way ANOVA followed by Tukey’s multiple comparisons).

Next, we examined whether TDI stimulation induces IL-33 release from the nuclei of nasal epithelial cells and whether SST inhibits it. Since TDI is insoluble in the culture medium and cannot be used for cell culture, a TDI-bound HSA (TDI-HSA) was prepared. After 1 h of TDI-HSA treatment, the percentage of cells expressing IL-33 in the nucleus decreased (Figure 5A,B). Pretreatment with SST significantly inhibited the release of IL-33 from the nuclei of epithelial cells upon TDI-HSA stimulation (Figure 5A,B).

## 4. Discussion

This study showed that SST, a Kampo medicine, suppressed symptoms associated with TDI-induced AR at least partially via inhibiting IL-33 release from the nuclei of nasal epithelial cells both in vivo and in vitro experiments.

The inflammatory response of the nasal mucosa consists of the following phases: IgE-mediated immediate mast cell response. Late-phase reaction and persistent allergic inflammation through mobilization of T cells expressing T_H_2 cytokines such as IL-4/5, which are growth factors for eosinophils, basophils, and eosinophils. Recent studies have revealed an additional important step: the expression and release in epithelial cells of cytokines such as IL-33, which regulates the T_H_2 cytokine response [17]. Critical roles of IL-33 in the AR development shown in human and experimental animals are functions to stimulate mast cells [2]. IL-33 protein is constantly localized in the nucleus of nasal epithelial cells in the normal condition and is released into NLF in response to allergen challenges, such as ragweed pollen [2]. Importantly, IL-33 knockout mice showed little AR response after sensitization with the allergen [2]. Elevated serum IL-33 levels have been reported in patients with seasonal AR, and a significant association between AR susceptibility and IL-33 polymorphisms has also been found [18]. However, it remains unclear whether IL-33 in the nucleus of nasal epithelial cells would be important for the TDI-induced AR. In this study, we revealed that the provocation with TDI in the TDI-sensitized rats significantly induced IL-33 release from the nucleus of nasal epithelial cells, and pretreatment with SST suppressed TDI-induced AR by inhibiting IL-33 release.

TDI is a recognized irritant to the human body and is one of the leading causes of occupational allergic disease in industrialized countries [19]. It has been reported that TDI-sensitized rodents showed similar features of nasal allergic diseases, including increased histamine levels and upregulation of histamine H1 receptors [20,21,22]. On the other hand, SST has been reported to ameliorate AR symptoms in human and animal/cellular models, and few mechanisms of the SST effect have been proposed [11,12,23,24,25,26,27,28]. SST inhibited eosinophilic infiltration of the nasal mucosa and improved symptoms of nasal obstruction in AR patients [12]. SST suppressed histamine release from mast cells in rats without affecting agonist binding to histamine H1 receptors [11]. Tanno et al. showed SST’s effect to inhibit basophil growth and differentiation in vitro and in vivo [23]. SST has also been reported to suppress T_H_2-type allergic reactions, affecting the differentiation of naive CD4+ T cells to Th1 or T_H_2 cells. SST decreased the expression of IL-4 mRNA, which is essential for T_H_2 cell development, and increased IFN-γ expression, which is important for Th1 cell development in mice [24,25]. Tanaka et al. also reported an effect of SST on suppressing the allergen-induced inflammatory process. SST decreased the synthesis of IgE and IL-10 in mononuclear cells of peripheral blood obtained from patients with perennial allergic rhinitis due to *Dermatophagoides farinae* [12]. We also previously reported that SST suppressed histamine-induced AR symptoms and substance-P/CGRP (calcitonin gene-related peptide) in NLF [26]. One electrophysiological study suggested that SST attenuated the secretion of water and electrolytes from the nasal glands induced by acetylcholine [27]. SST seems to affect the step of the infiltration of mast cells and eosinophils into the nasal mucosa in AR development [28,29]. Nakano et al. showed that SST suppressed phorbol ester-induced increase of IL-33 and histamine H1 receptors in cultured Swiss 3T3 and HeLa Cells [30]. In addition to these reported functions of SST, our study would raise a possible mechanism underlying how SST ameliorates symptoms of AR patients as inhibition of IL-33 release from nasal epithelial cells stimulated by allergens. However, it is still unclear how SST blocks the release of IL-33 from epithelial cells.

IL-33 is synthesized as the full-length form and localized in the nucleus in epithelial cells. The full-length IL-33 is cleaved to generate a high-activity form when cells are exposed to environmental aeroallergens. That process has an important function in the rapid and efficient induction of allergic type 2 responses [31]. The step of the cleavage of IL-33 performed by proteases contained in allergens and/or intracellular proteases such as calpain has been reported to be essential for IL-33 release. Allergen-induced secretion of IL-33 from epithelial cells was reported to be mediated by redox-dependent activation of EGFR signaling pathways induced by activation of NADPH oxidase isoform (DUOX1), which in turn activates a protease calpain-2 [32]. Interestingly, extracts and components of herbs consisting of SST have been shown to inhibit EGFR signaling. Extract of Ephedra herb, a component of SST, strongly suppressed EGFR protein expression within several hours [33]. Trans-cinnamaldehyde contained in Cinnamon extract, which is also a component of SST, is reported to inhibit EGFR activation in vitro [34]. 8-gingerol contained in ginger extract had a role as an inhibitor of EGFR [35]. Although the molecular mechanism of the inhibitory effect of SST on IL-33 release from the epithelial cell nucleus needs to be clarified in the future, suppression of EGFR signaling may be one of the mechanisms.

## 5. Conclusions

Recent basic research on herbal medicines has revealed many useful actions and their molecular mechanisms. Since IL-33 release from nasal epithelial cells is considered to be a common mechanism of AR exacerbation, the use of SST is highly promising not only for AR caused by TDI but also for AR caused by other allergens. A limitation of this study is that it is not yet clear how SST blocked IL-33 release from epithelial cells. Further studies are needed to clarify its molecular mechanism. It is likely that SST will be used more and more in clinical practice in the future because of the advantages of herbal medicine, which has fewer side effects and has a combined action.

## Data Availability

The data used and/or analyzed during the current study are available from the corresponding author upon reasonable request.
